# Supplementary figures and images for: Transcriptional profiling reveals potential involvement of microvillous TRPM5-expressing cells in viral infection of the olfactory epithelium
Source: BMC Genomics. 2021 Mar 30;22:224. doi: 10.1186/s12864-021-07528-y (PMC8007386; doi:10.1186/s12864-021-07528-y)

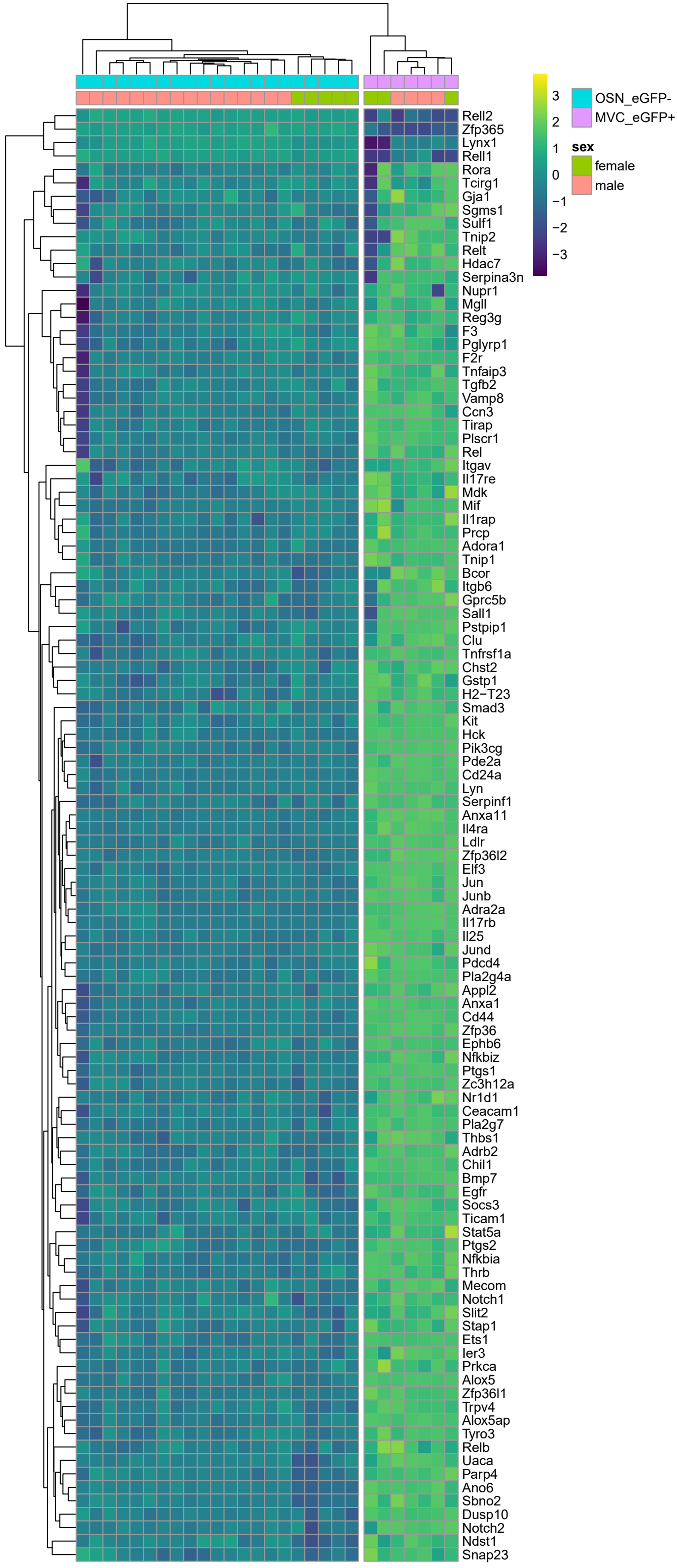

Supplement: Supplementary file 1 — Additional file 1. [file 12864_2021_7528_MOESM1_ESM.zip › Fig 3 fig sup 1 MVCvOSN_GFP-_inflam.pdf]

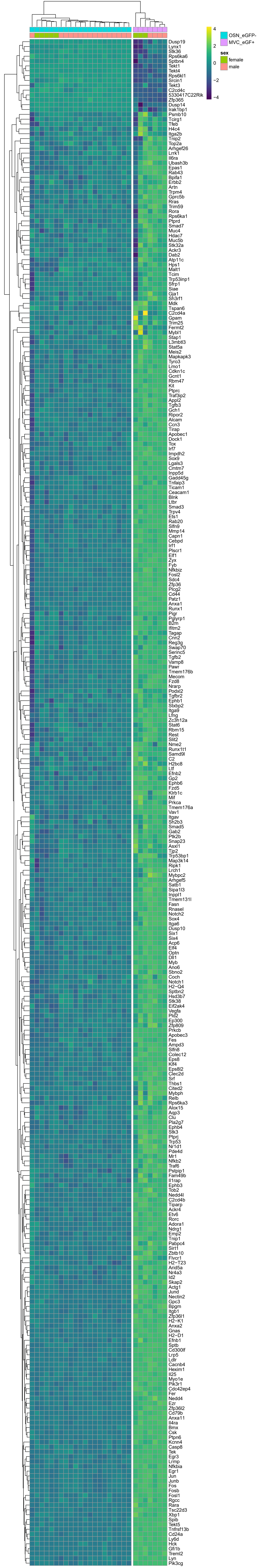

Supplement: Supplementary file 1 — Additional file 1. [file 12864_2021_7528_MOESM1_ESM.zip › Fig 3 fig sup 2 MVCvOSN_GFP-_immune.pdf]

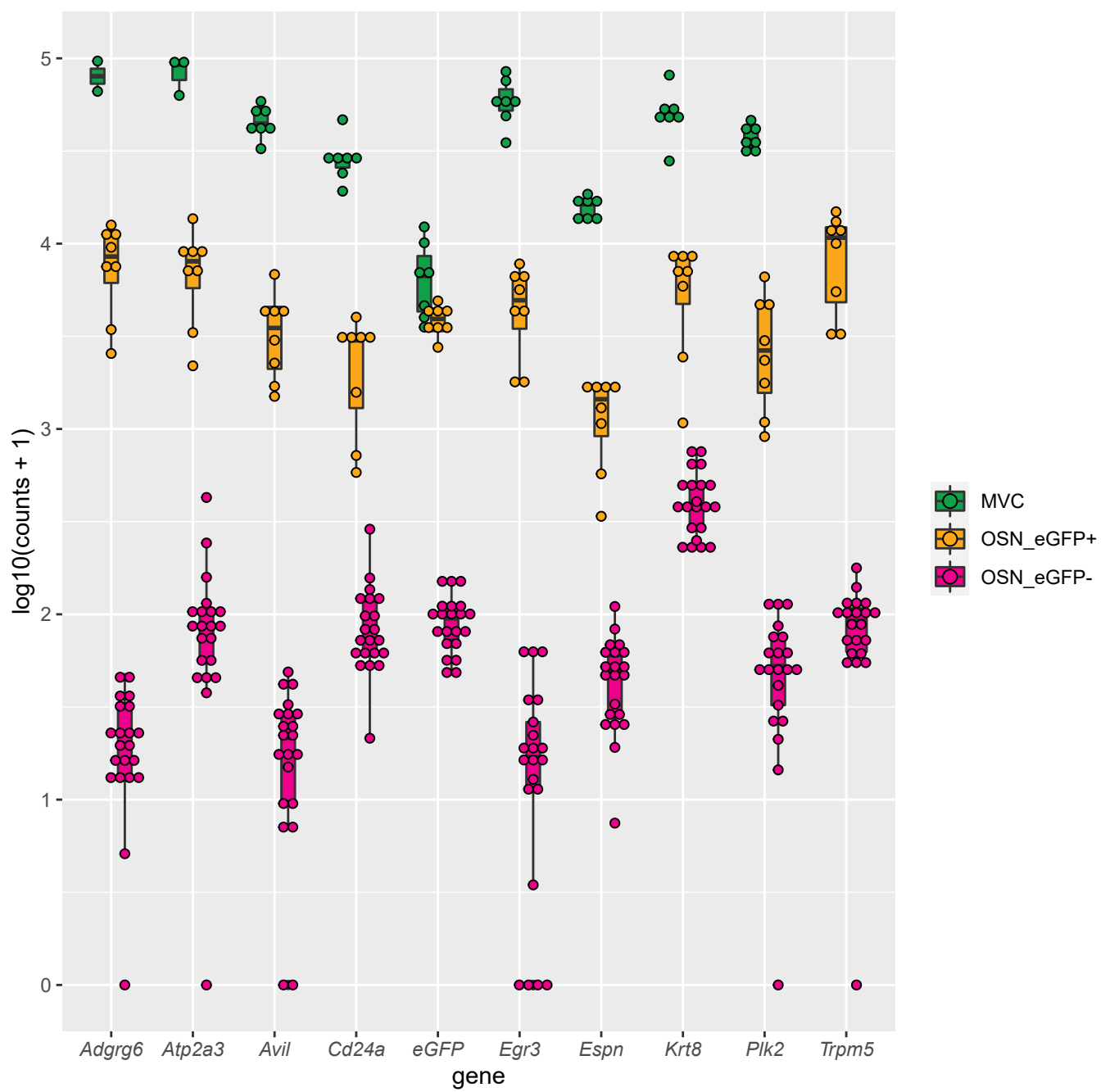

Supplement: Supplementary file 1 — Additional file 1. [file 12864_2021_7528_MOESM1_ESM.zip › Fig 4 fig sup 4 boxplot_withOSNGFP.pdf]

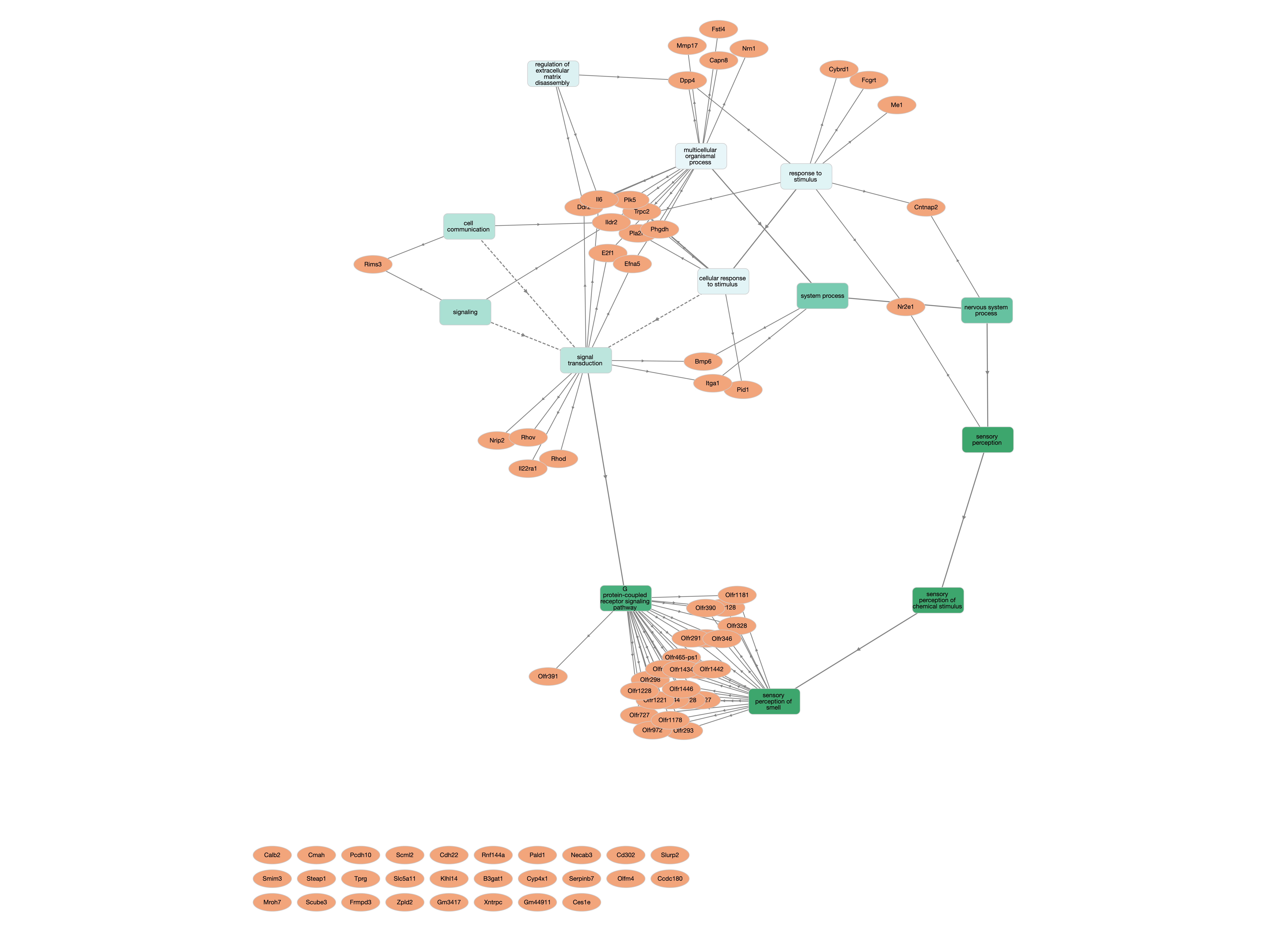

Supplement: Supplementary file 1 — Additional file 1. [file 12864_2021_7528_MOESM1_ESM.zip › Fig 4 fig supp 6 GOnet analysis.png]

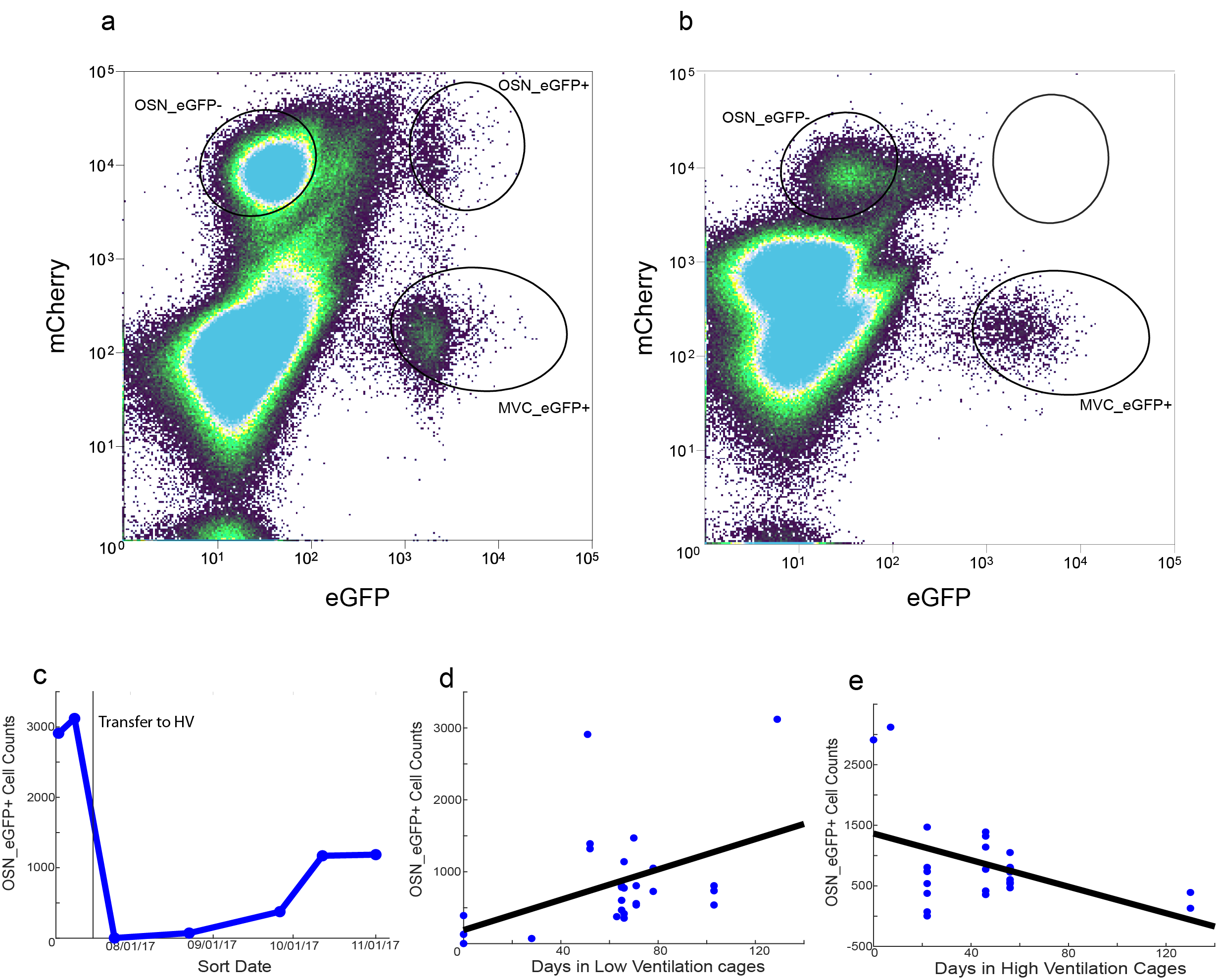

Supplement: Supplementary file 1 — Additional file 1. [file 12864_2021_7528_MOESM1_ESM.zip › Fig1_supp1_FACSadded_V2.png]

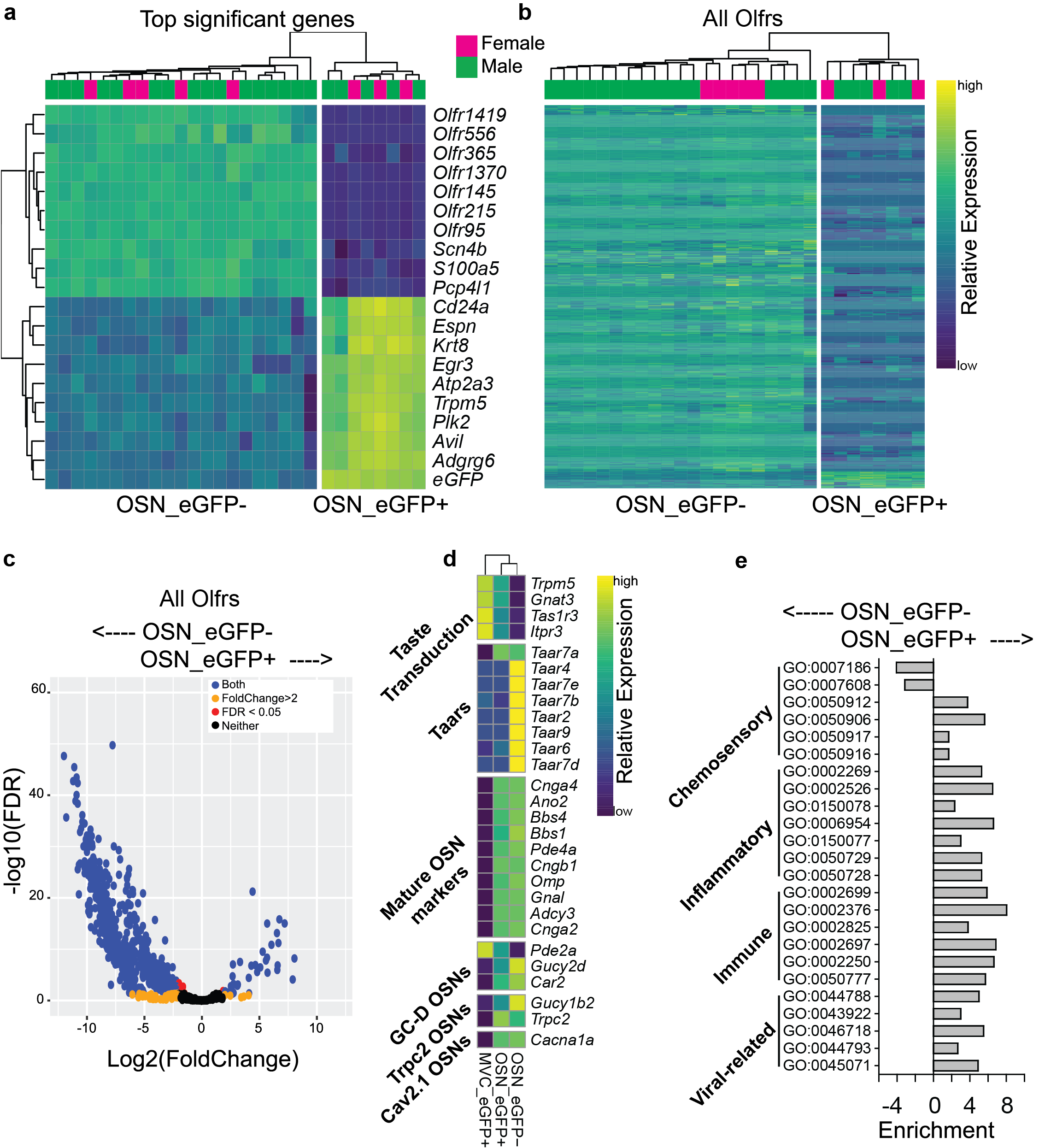

Supplement: Supplementary file 1 — Additional file 1. [file 12864_2021_7528_MOESM1_ESM.zip › Fig4_fig sup 2 OSNS_V6.png]

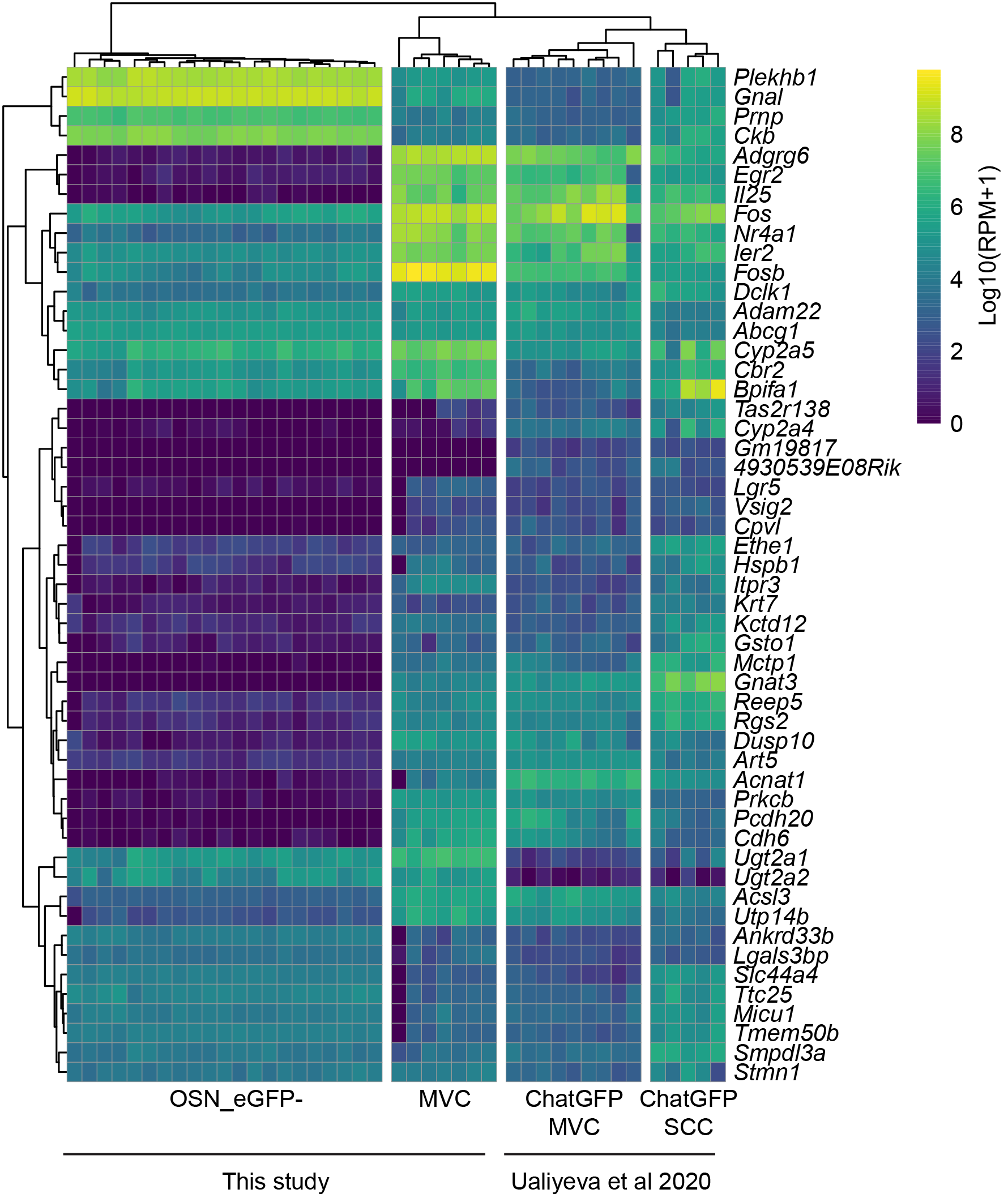

Supplement: Supplementary file 1 — Additional file 1. [file 12864_2021_7528_MOESM1_ESM.zip › Figure 2 - fig supp 4 MVCcomparisons_V2.png]

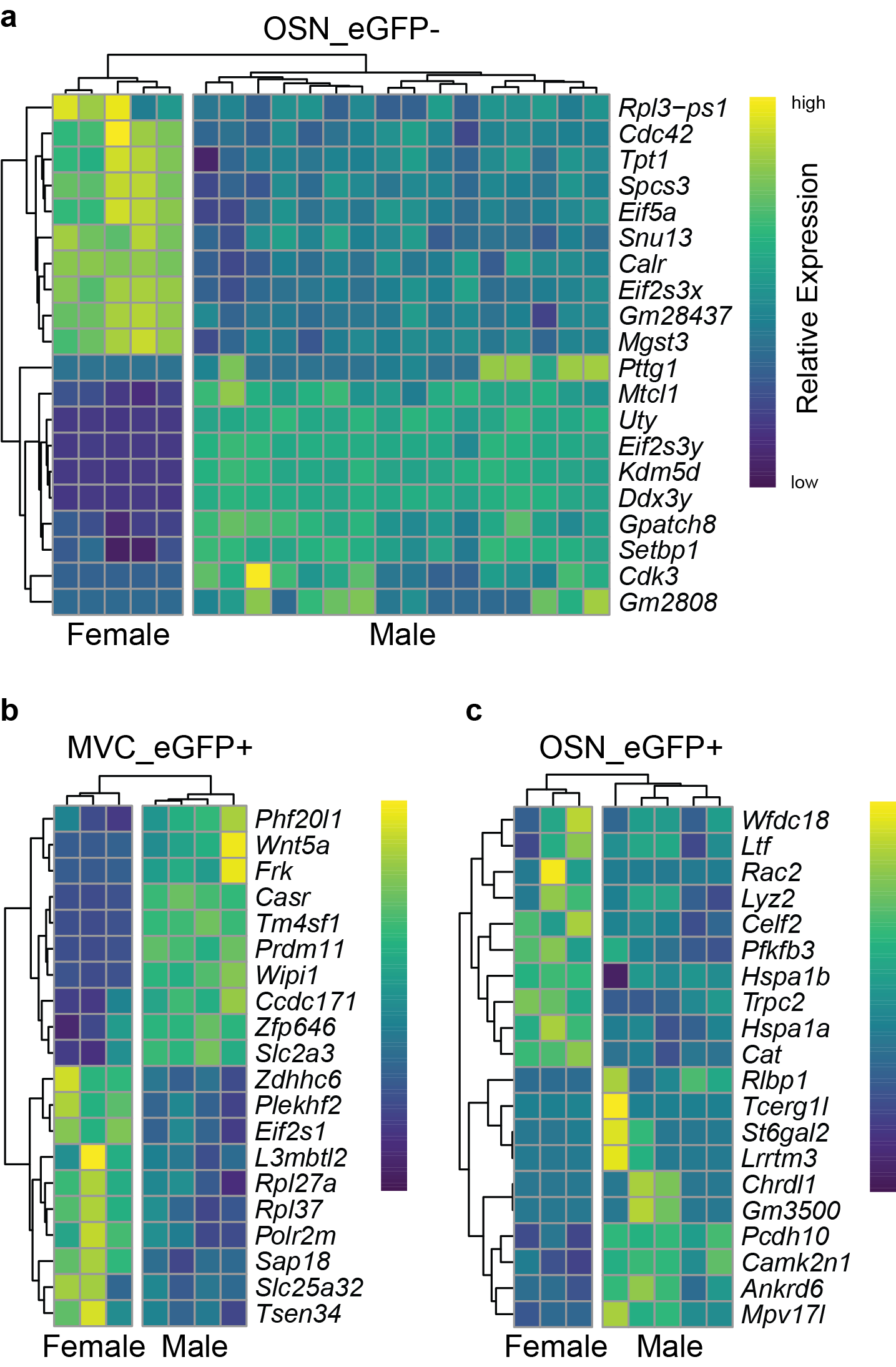

Supplement: Supplementary file 1 — Additional file 1. [file 12864_2021_7528_MOESM1_ESM.zip › Figure 4-fig sup 7_sexdiff.png]

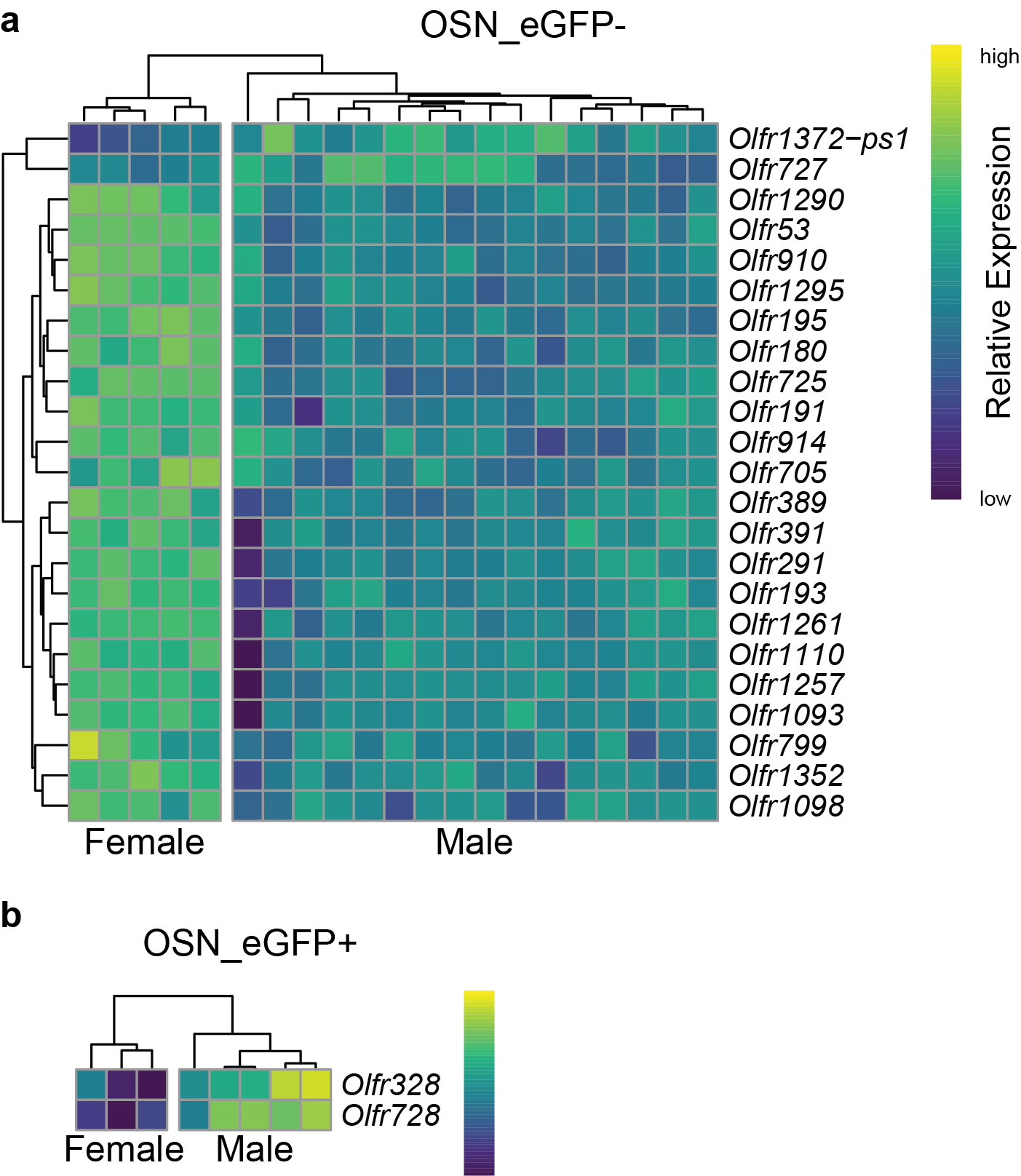

Supplement: Supplementary file 1 — Additional file 1. [file 12864_2021_7528_MOESM1_ESM.zip › Figure 4-fig sup 8_sexdiff_olfrs.png]
